# The Expression and Characterization of Functionally Active Soluble CD83 by *Pichia pastoris* Using High-Density Fermentation

**DOI:** 10.1371/journal.pone.0089264

**Published:** 2014-02-20

**Authors:** Yugang Guo, Rui Li, Xiaoping Song, Yongjun Zhong, Chenguang Wang, Hao Jia, Lidan Wu, Dong Wang, Fang Fang, Jiajia Ma, Wenyao Kang, Jie Sun, Zhigang Tian, Weihua Xiao

**Affiliations:** 1 Hefei National Laboratory for Physical Sciences at Microscale, Innovation Center for Cell Biology, School of Life Sciences, University of Science and Technology of China, Hefei, China; 2 Center of Medical Biotechnology of Anhui Province, University of Science and Technology of China, Hefei, China; 3 Department of Pharmacy, Anhui Medical College, Hefei, China; Université Libre de Bruxelles, Belgium

## Abstract

CD83 is a highly glycosylated type I transmembrane glycoprotein that belongs to the immunoglobulin superfamily. CD83 is upregulated during dendritic cell (DC) maturation, which is critical for the initiation of adaptive immune responses. The soluble isoform of CD83 (sCD83) is encoded by alternative splicing from full-length CD83 mRNA and inhibits DC maturation, which suggests that sCD83 acts as a potential immune suppressor. In this study, we developed a sound strategy to express functional sCD83 from *Pichia pastoris* in extremely high-density fermentation. Purified sCD83 was expressed as a monomer at a yield of more than 200 mg/L and contained N-linked glycosylation sites that were characterized by PNGase F digestion. *In vitro* tests indicated that recombinant sCD83 bound to its putative counterpart on monocytes and specifically blocked the binding of anti-CD83 antibodies to cell surface CD83 on DCs. Moreover, sCD83 from yeast significantly suppressed ConA-stimulated PBMC proliferation. Therefore, sCD83 that was expressed from the *P. pastoris* was functionally active and may be used for *in vivo* and *in vitro* studies as well as future clinical applications.

## Introduction

CD83 is a highly glycosylated type I transmembrane glycoprotein with 186 amino acids. CD83 is upregulated during dendritic cell (DC) maturation and plays an essential role in the initiation of adaptive immune responses [1], [2]. Recent studies have demonstrated that CD83 is expressed in most immune cells and plays an important role in regulating innate and adaptive immune responses, including mediating the activation of T cells by DCs [3]–[8], controlling the thymic maturation and activation of CD4^+^ single-positive lymphocytes, and maintaining the homeostasis of B lymphocytes [9], [10]. Therefore, CD83 has a wide range of immune regulation functions.

In addition to the transmembrane form of CD83, an alternative isoform of soluble CD83 (sCD83), which results from the alternative splicing of full-length CD83, had been found in the serum of healthy adults and patients with leukemia, malignant tumors, and rheumatoid arthritis [8], [11]–[14]. Impressively, recombinant sCD83 from *Escherichia coli* (*E. coli*) exhibited significant immunosuppressive properties, such as inhibiting the development of cellular anti-tumor immunity [15]–[17], inducing allograft tolerance, and suppressing autoimmune disorders in the experimental autoimmune encephalomyelitis (EAE) model [8], [18]–[21]. Therefore, sCD83 represents a potential therapeutic tool and immune regulation target.

However, despite the intense research on CD83 during the last decade, CD83 remains an orphic ligand. A CD83 receptor has not been defined and the respective involved signal transduction pathways have not been fully elucidated. Nevertheless, several indicators suggest possible locations of the CD83 receptor, including mouse B cells, human DCs, monocytes, and CD8^+^ T cells [11], [22], [23]. Recently, a possible CD83 receptor on human monocytes was demonstrated to control the activation of NF-κB and was involved in the regulation of PGE2 production [24]. However, the enigmatic nature of this possible CD83 receptor significantly slowed down and impeded further studies on CD83 [25]. Therefore, understanding the function of sCD83 is a precondition for the identification of a CD83 receptor.

Techniques that enable the production of sCD83 on a large scale may be favored to study a putative CD83 receptor, the mechanisms of sCD83 immunosuppression, and the potential immunotherapeutic applications of sCD83. However, the large-scale production of bioactive sCD83 using traditional methods is challenging. Previously, recombinant sCD83 was expressed by *E. coli* and purified using anion-exchange chromatography [26]. However, this study was affected by several common shortcomings of prokaryotic expression systems, such as inclusion bodies, non-glycosylation, and high endotoxin contamination. Therefore, HEK293T cells were recently investigated as a mammalian cell expression system to express sCD83 [27]. However, the glycosylation of sCD83 was far from homogeneous, which led to different isoforms that ranged from 15 kDa to 45 kDa. In addition, this system experienced common limitations of mammalian expression systems, which are expensive, time-consuming, and inefficient. Recently, human sCD83 that was fused to hIgG1 Fc was expressed by *P. pastoris* [28]. However, the degradation in this system limited the large-scale production of sCD83, and the fusion of the Fc region to sCD83 may affect immune function in subsequent CD83 research mainly due to the wide expression and multiple functions of the Fc receptor in the human immune system. A commercial recombinant sCD83/Fc fusion protein that was expressed by insect cells is available from R&D Systems but has similar problems as mammalian expression systems.

In this study, we developed a simple and robust fed-batch fermentation process for the production of recombinant human sCD83 using *P. pastoris* in basal salt medium at a high cell density. The sCD83 yield reached at least 200 mg/L by fermentation, and over 95% purity was achieved with common His-Select affinity chromatography and size-exclusion chromatography. Yeast-expressed sCD83 is mainly found as a monomer, which is consistent with sCD83 from HEK293 cells [27]. Further studies have demonstrated that sCD83 from yeast may have the same functional structure as natural human surface CD83 and can interact with the CD83 receptor. Moreover, a functional analysis revealed the significant inhibition of human peripheral blood mononuclear cell (PBMC) proliferation by sCD83. These results suggest that fermentation in *P. pastoris* provides a sound strategy for large-scale recombinant sCD83 production, which may be used in basic research and clinical applications.

## Materials and Methods

### Ethics Statement

Healthy human peripheral blood was obtained from the Blood Centre of Anhui Province (Hefei, China) and all participants provided written informed consent. The study was approved by the Ethics Committee of the University of Science and Technology of China.

### Strains, Plasmids, and Media

The pPIC9K vector, the *E. coli* strain DH5α, and the *P. pastoris* strain GS115 were obtained from Invitrogen. The media and protocols for *P. pastoris* were used according to the *Pichia* Expression Manual and *Pichia* Fermentation Process Guidelines of Invitrogen.

### Vector Construction and Transformation

According to the cDNA sequence of sCD83 (RefSeq: NM_004233.2), two primers were designed to amplify the coding sequence of sCD83. The CD83-Fw-*Xho* I primer (5′- TCTCTCGAGAAAAGAACGCCGGAGGTGAAGGTGGCTT.GC-3′) contained an *Xho* I restriction site (underlined), whereas the CD83-his-Rv-*EcoR* I primer (5′-CTGAATTCTCAATGGTGATGGTGATGATGAATCTCCGCTCTGTATTTC-3′) contained an *Eco*R I restriction site (underlined). For easy detection and convenient purification, a 6xHis coding sequence was added to the 5′-end of the reverse primer of sCD83.

**Figure 1:**
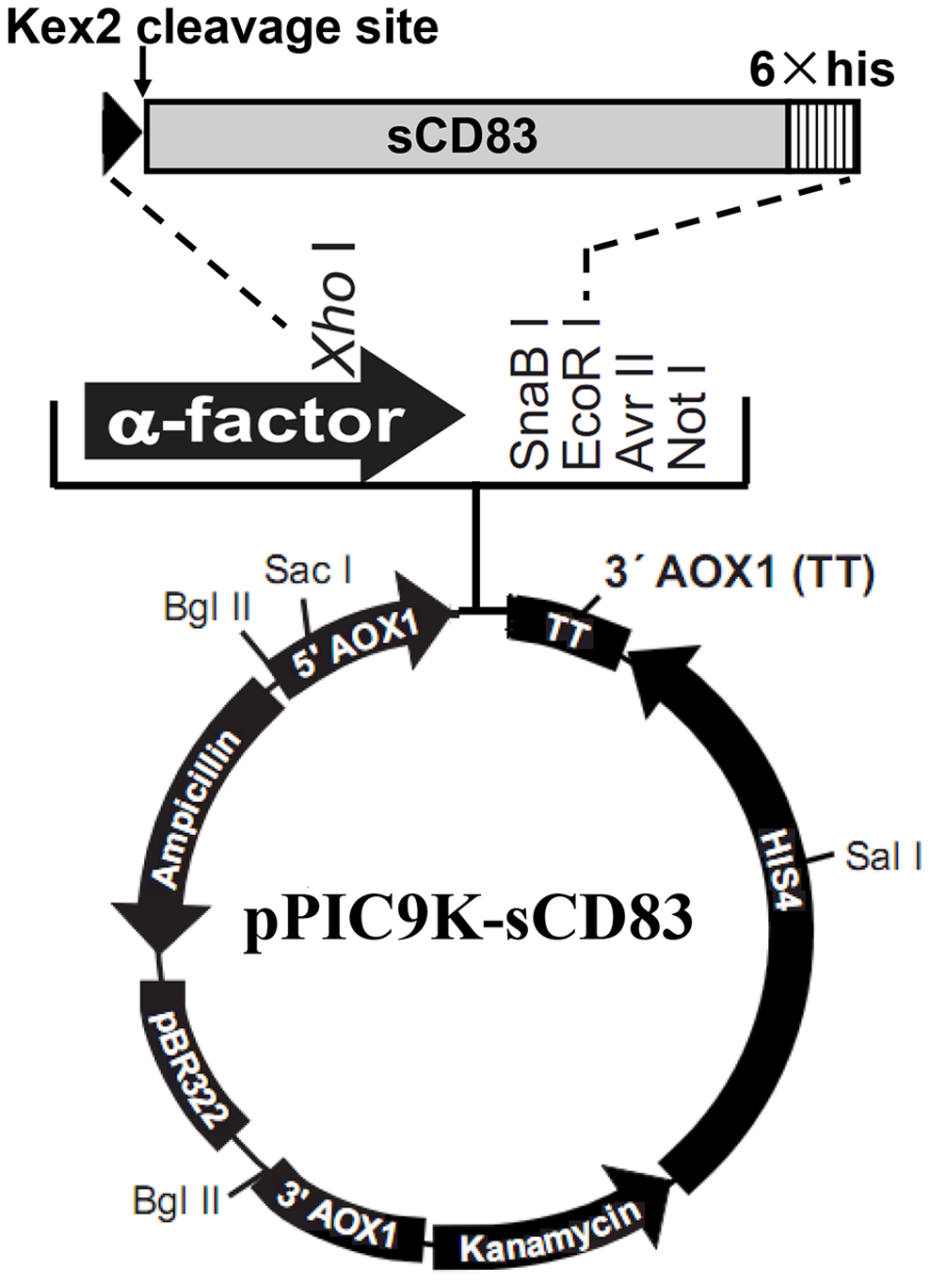
A schematic genetic map of the sCD83 expression vector. The sCD83 gene was inserted between the *Xho* I and *Eco*R I sites in pPIC9K and in-frame with the Kex2 cleavage site in the sequence of the α-factor secretion signal to create the expression vector pPIC9K-sCD83.

Using the human cDNA clone pCMV6-XL4-CD83 (OriGene) as the template, the sCD83 gene was obtained by polymerase chain reaction (PCR) and inserted between the *Xho* I and *EcoR* I sites in pPIC9K and in-frame with the Kex2 cleavage site in the sequence of the α-factor secretion signal to create the expression vector pPIC9K-sCD83. The insertion sequence in the recombinant vector pPIC9K-sCD83 was confirmed through DNA sequencing, and the schema for cloning is shown in Fig. 1.

Linearized vectors by *Sal* I were transformed into *P. pastoris* GS115 as previously described, which resulted in many GS115/sCD83 colonies that were screened using MD plates. To obtain high-expressing colonies, these colonies were further screened on yeast extract peptone dextrose (YPD) plates that contained different concentrations of G418 according to the *Pichia* Expression Manual of Invitrogen.

### Induced Expression in Shake Flasks

Several colonies of transformants were randomly selected from the YPD plates that contained 1.0 mg/ml G418 (YPDG). The colonies were cultured in 50 ml of buffered glycerol-complex (BMGY) medium at 30°C. After 24 h, the yeast was centrifuged for 5 min at a speed of 1,500 *g* to be pelleted and resuspended in buffered methanol-complex (BMMY) medium. The yeast was inducted continually for 72 h during which 100% methanol was added every day to maintain a constant concentration of 0.5%. After 72 h, the yeast was centrifuged for 10 min at a speed of 10,000* g* and the supernatants were collected. Western blot analysis was used to analyze the supernatants with anti-His tag monoclonal antibodies (Abmart).

To obtain the optimal conditions for expressing sCD83 from a selected colony through fermentation, several factors that could affect the expression level of the protein were tested in flasks. These factors included temperature (20–30°C) and pH (4.0–6.0). The experiments with these conditions were performed in triplicate. Protein production was analyzed using Western blot analysis.

### High-density Fermentation

The fermentation process for sCD83 was performed according to the *Pichia* Fermentation Process Guidelines of Invitrogen. Briefly, the stock of the transformed *P. pastoris* strain was recovered on a MD plate. Four colonies were selected and cultivated at 30°C and 250 rpm for approximately 20 h, and the OD_600_ reached 2–6. These colonies were used as the primary seeds. One of these seeds was chosen and cultivated for another 20 h in a 1,000-ml shake flask that contained 200 ml of BMGY medium, and the OD_600_ reached 2–6. This culture served as the secondary seed. A 14-L fermenter (New Brunswick Scientific, BioFlo 115) that contained 6 L of BSM, which was supplemented with 6.0 ml/L of PTM1 solution, was inoculated with 200 ml of the secondary seed. The temperature was controlled at 30°C, and the dissolved oxygen (DO) was maintained at above 90% air saturation. The agitation speed was initially set at 600 rpm, and the pH was maintained at 5.0 by adding 28% ammonium hydroxide. Glycerol feeding was initiated when the DO increased rapidly due to the exhaustion of the carbon source. The feeding medium contained 50% glycerol and 12 ml/L of PTM1 solution. The glycerol feeding rate was 18 ml/h/L for another 2–4 h until the wet weight of the culture reached 180 g/L. When glycerol feeding was ended, 100% methanol with 12 ml/L of PTM1 solution was added to induce the expression of sCD83. The feeding rate of methanol was adjusted as designed. During the methanol induction period, the DO was set to 20%–40% air saturation and controlled by agitation or the addition of air. Pure oxygen was supplemented when necessary to control the DO. The wet cell weight (WCW) and the OD_600_ were determined every 6 h from the beginning of the fed-batch phase. To harvest more supernatants, 2 L of fermentation culture was drawn from the fermenter at 48 and 72 h after induction.

### The Purification of sCD83

The pH of the fermentation culture was adjusted to 8.0 for nickel affinity gel purification before centrifugation. The crude supernatant was collected and further processed using a hollow fiber microfiltration system (FlexStand microfiltration system, GE Healthcare). The clarified supernatant was loaded onto a nickel affinity column with a flow speed of 1.2 L/h. The column was first washed with 5 column volumes of binding buffer (20 mM Tris-HCl, pH 8.0; 0.3 M NaCl) and then washed with another 5 column volumes of wash buffer (the binding buffer contained 20 mM imidazole). sCD83 was eluted with elution buffer (the binding buffer contained 250 mM imidazole), and the eluate was concentrated by tangential flow filtration with a 10-kDa filter (TFF Labscale, Millipore). The concentrated eluate was further purified by size-exclusion chromatography (S-200 HR, GE Healthcare) on an AKTA Explorer System (GE Healthcare) and eluted with phosphate-buffered saline (PBS) at a flow speed of 1 ml/min. Each peak of eluted fractions was collected and analyzed by sodium dodecyl sulfate polyacrylamide gel electrophoresis (SDS-PAGE) and stored at −80°C.

### The Deglycosylation of sCD83 Produced by *P. pastoris*

Approximately 20 µg of purified sCD83 was incubated with 2.0 IU of PNGase F (New England Biolabs) at 37°C for 3 h according to the manufacturer’s instructions. Then, the reaction mixture was analyzed using SDS-PAGE and Western blotting under reducing conditions.

### SDS-PAGE and Western Blotting

The samples were separated using 15% PAGE under reduced and non-reduced conditions, and the gel was stained with Coomassie blue. For Western blotting, the supernatant was separated by SDS-PAGE and then transferred to a nitrocellulose membrane (Whatman GmbH, Germany) for 20 min at 18 V in transfer buffer (25 mM Tris, 192 mM glycine, and 10% methanol). After blocking the membrane with 5% skim milk in Tris-buffered saline (TBS, 10×TBS: 0.2 M Tris; 1.5 M NaCl) that contained 0.1% Tween-20 (TBST) at room temperature for 1 h, the membrane was incubated with the indicated antibodies for 1–2 h at room temperature. Then, the membrane was washed 5 times with TBST and incubated with the corresponding secondary antibody IgG-HRP conjugates. After washing the membrane with TBST, the protein was detected using an EZ-ECL Chemiluminescence detection kit.

### Cell Culture

Human PBMCs were isolated from the peripheral blood of healthy donors by Ficoll-Hypaque density gradient centrifugation (Solarbio). Subsequent Percoll density gradient centrifugation (GE Healthcare) was performed for monocyte separation as previously reported [29]. Human monocyte-derived DCs were generated by culturing the cells with rhGM-CSF (50 ng/mL, PeproTech) and rhIL-4 (50 ng/mL, PeproTech) in RPMI 1640 medium (RPMI 1640 with 2 mM glutamine, 25 mM HEPES, 100 U/ml penicillin, and 100 mg/ml streptomycin) that contained 10% fetal bovine sera (HyClone) at 37°C in a CO_2_ (5%) incubator for 5 days. To obtain mature DCs, the cells were cultured in the same cytokine mixtures in the presence of LPS (50 ng/mL, Sigma-Aldrich) for an additional 2 days.

### Biological Activity Assay

To detect the specificity of sCD83 and the polyclonal antibody from sCD83 immune rabbit serum (sCD83 pAb, preparation by GenScript, Nanjing), antibody competition was performed as follows: mature DCs (5×10^5^) were harvested and coated with antibodies for typical surface marker molecules of DCs (BD Biosciences) in the presence of sCD83 (0–10 µg) or polyclonal antibodies (0–30 µg).

To detect the CD83 counterpart, PBMCs were isolated from the peripheral blood of healthy adult volunteers using consecutive Ficoll-Hypaque density gradient centrifugation as previously reported [29]. The PBMCs were incubated with sCD83 (20 µg/mL) or an equal concentration of bovine serum albumin (BSA) for 1 h at room temperature in PBS that contained 3% mouse serum to block the Fc receptor. After the unbound proteins were removed by washing with PBS, either PE-conjugated mouse anti-human CD83 antibodies (BD Bioscience) or mouse anti-His followed by FITC-anti-mouse IgG (BioLegend) were added. FITC or PE-labeled monoclonal antibodies against CD3 or CD14 (BD Bioscience) were added, and a 2-color acquisition protocol was used for flow cytometry analysis.

To detect the immunosuppressive activity of sCD83, ConA-stimulated PBMC proliferation was used as previously reported [28]. PBMCs were stained with 1.5 µM carboxyfluorescein diacetate succinimidyl ester (CFSE, Invitrogen), seeded at a density of 4×10^5^ cells/well in 48-well plates, and cultured for 3–6 days after stimulation with ConA (5 µg/mL, Sigma) in the presence of sCD83 (0–20 µg/mL). The cells were detected using a FACSCalibur flow cytometer (BD Biosciences), and the data were analyzed using FlowJo (Tree Star).

To determine whether NF-κB activation was mediated by sCD83, the isolated human PBMCs that were stimulated by sCD83 (10 µg/mL) were cultured in RPMI 1640 medium that contained 10% fetal bovine sera at 37°C for 12 h. The PBMCs were collected and lysed to detect the activation of the NF-κB pathway. The anti-p-p65 Ser276 antibody was from Santa Cruz. The anti-p65 antibody was from Epitomics. The anti-p-IκBα Ser32 antibody and the anti-IκBα antibody were obtained from Cell Signaling.

## Results

### Vector Construction and the Expression of sCD83 in *P. pastoris*

**Figure 2:**
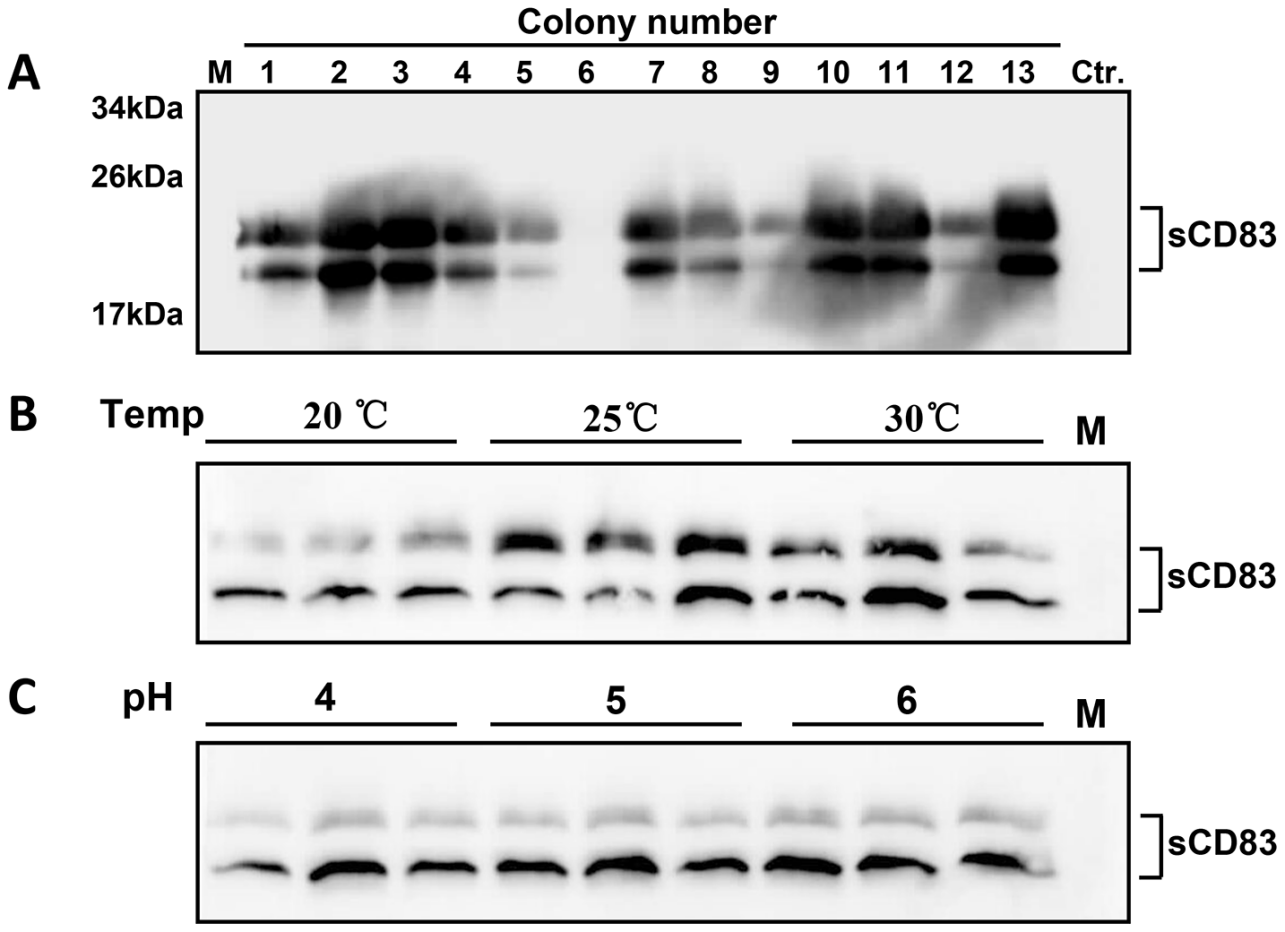
The screening and optimization of sCD83 expression in flasks. (A) The screening of high-expressing colonies of sCD83 from YPDG plates. 1–13: the colony number, Ctr. : the GS115/pPIC9K control. (B) The optimization of the induction temperature in flasks. (C) The optimization of the induction pH in flasks. Each experiment with these conditions was performed in triplicate.

Full-length CD83 is a 45-kDa highly glycosylated transmembrane protein that consists of 186 amino acids and extracellular, transmembrane, and intracellular domains. The sCD83 coding sequence was inserted into *Xho* I and *Eco*R I restriction enzyme sites of pPIC9K to generate pPIC9K-sCD83. The expression vector pPIC9K-sCD83 was linearized with *Sal* I and transformed into the *P. pastoris* strain GS115 via electroporation. The colonies that expressed sCD83 were examined using Western blot analysis and a mouse anti-His tag monoclonal antibody. The results indicated that these colonies secreted different levels of sCD83 (Fig. 2A). One of the best secreting colonies was cultured in a flask and stored at −80°C as seeds for fermentation.

Process characterization is a systematic investigation of the impact of operating parameters on expression levels. To determine the optimal temperature and pH for induction, a stock of sCD83 was cultured in a shake flask that contained BMGY medium and induced in BMMY medium in experiments that tested each single parameter. The expression levels of the target protein were monitored using Western blot analysis and the mouse anti-His tag monoclonal antibody after the proteins were separated by 15% SDS-PAGE under reduced conditions. Figure 2B shows that the expression levels of sCD83 were not significantly affected by changes in temperature that ranged from 25–30°C; however, reduced levels of expression were observed at 20°C. In addition, we found that the pH of the culture had little effect on the expression levels (Fig. 2C).

### Large-scale Fermentation

**Figure 3:**
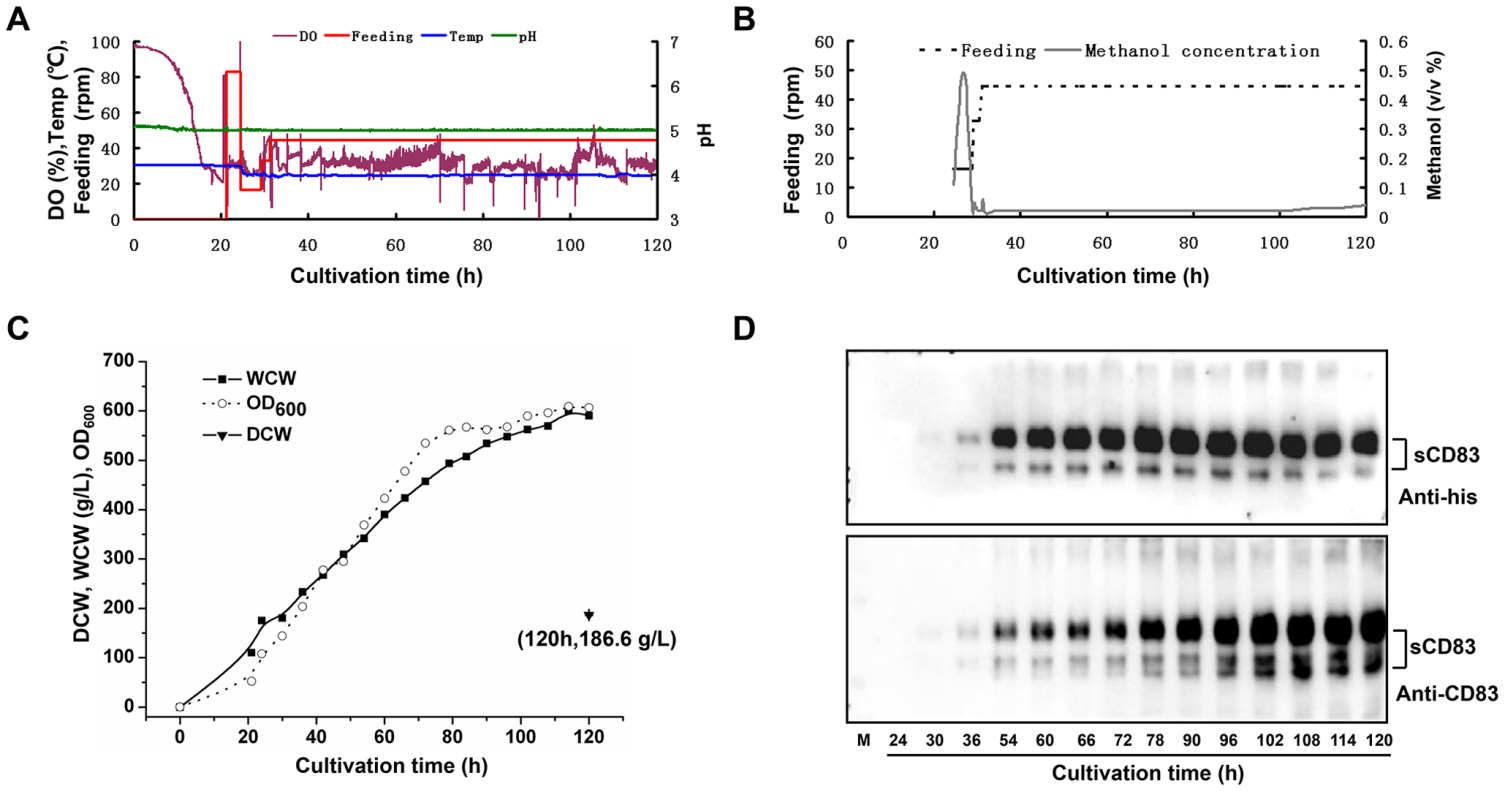
The fermentation of sCD83 in *P. pastoris*. (A) The plotted parameters show the DO, the pH, the temperature, and the feeding speed during fermentation. (B) Methanol feeding and the concentration during fermentation. (C) The biomass increases in DCW, WCW, and OD_600_ during induction. (D) Western blot analysis of the time period for expression during fermentation.

To obtain large amounts of recombinant sCD83, a simple but robust fermentation procedure in a 14-L fermenter was applied. The initial batch was grown in defined BSM medium that contained 40 g/L of glycerol. An increase in DO due to carbon source limitation was detected at approximately 20 h of cultivation; therefore, the glycerol fed-batch process was initiated for another 3 h until the biomass level (WCW) reached 175.3 g/L, at which time induction by methanol was initiated. During the first 2–3 h of the induction phase, the DO value was erratic until the culture adapted to methanol. After adaptation, a constant feeding rate was maintained throughout the remainder of the fermentation. The typical plotted parameters of the fermentation are shown in Fig. 3A. We maintained the methanol concentration at a constant low level to sustain limited growth after adaptation (Fig. 3B). At the end of the fermentation, the WCW of the culture reached approximately 600 g/L, which corresponded to a dry cell weight (DCW) of 180 g/L (Fig. 3C). The WCW was increased during the induction phase; however, the expression levels of sCD83 accumulated progressively during the first 80 h after methanol induction and were subsequently maintained or declined (Fig. 3D). The expression levels of sCD83 in the supernatants were greater than 200 mg/L by gel scanning.

### Purification and Characterization of sCD83

**Figure 4:**
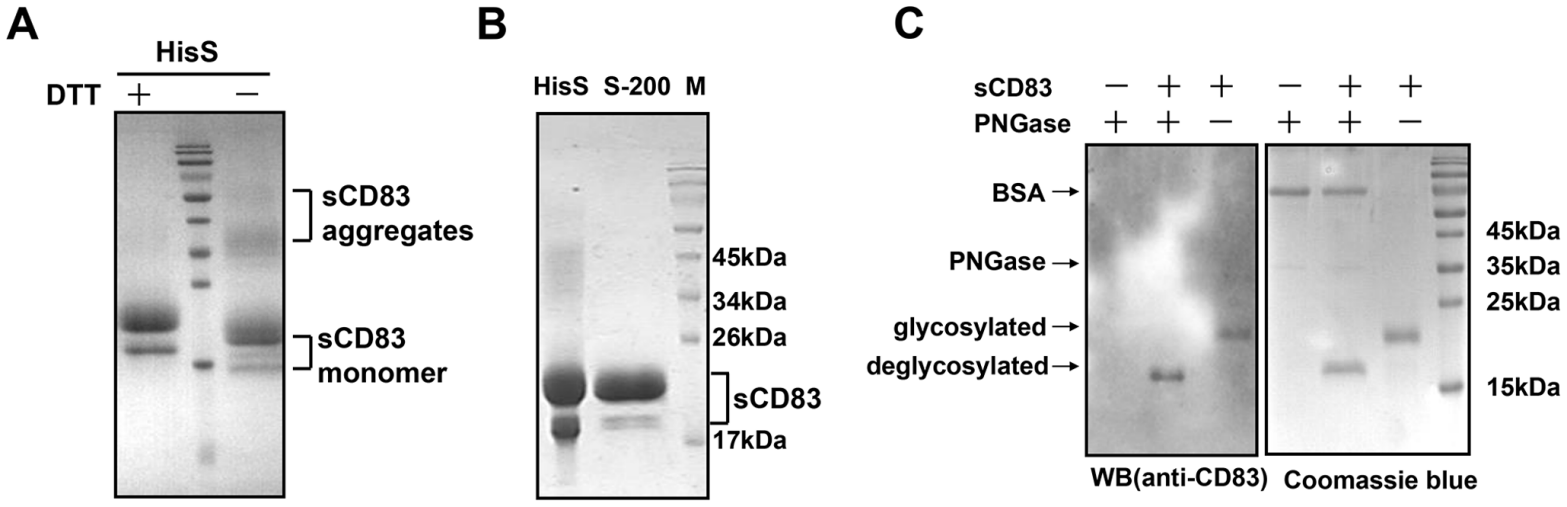
The purification and characterization of sCD83. (A) The isolation of sCD83 by HisS from the fermentation supernatant. HisS: His-Select chromatography. (B) The further purification of sCD83 by S-200. S-200: size-exclusion chromatography S-200 HR, M: pre-stained protein molecular marker. (C) Approximately 20 µg of sCD83 was incubated with 2.0 IU of PNGase F according to the manufacturer’s instructions. The reaction mixture was analyzed in 15% SDS-PAGE and stained with Coomassie blue. Western blotting was performed using anti-CD83 polyclonal antibodies under reducing conditions.

The clarified supernatant was obtained by centrifugation and filtration after fermentation and was directly loaded onto a His-Select column to capture the sCD83 protein, which was further purified using an S-200 HR column. We found that sCD83 from yeast was mainly expressed as a monomer and partially as a dimer (Fig. 4A), which was consistent with sCD83 expressed from HEK293T cells but not from *E. coli* [27], [30]. The purity of sCD83 reached 95% after the two-step purification procedure (Fig. 4B), and the final yields were approximately 100 mg/L. The bacterial endotoxin level of purified sCD83 was <0.1 EU/µg protein according to the limulus amebocyte lysate assay.

We found that sCD83 appeared as two close bands in 15% SDS-PAGE. Therefore, we speculated that these two bands were due to glycosylation, which is the most common post-translational modification of recombinant proteins that are expressed in yeast. Glycosylation site prediction software (http://expasy.org/tools/) indicated that 3 potential N-glycosylation sites were present in sCD83, which corresponded to the results of a previous report. The molecular mass of sCD83 that was treated with PNGase F was reduced by approximately 3–4 kDa (Fig. 4C), which indicates that sCD83 that is expressed in *P. pastoris* is glycosylated.

### sCD83 Specificity Assay

**Figure 5:**
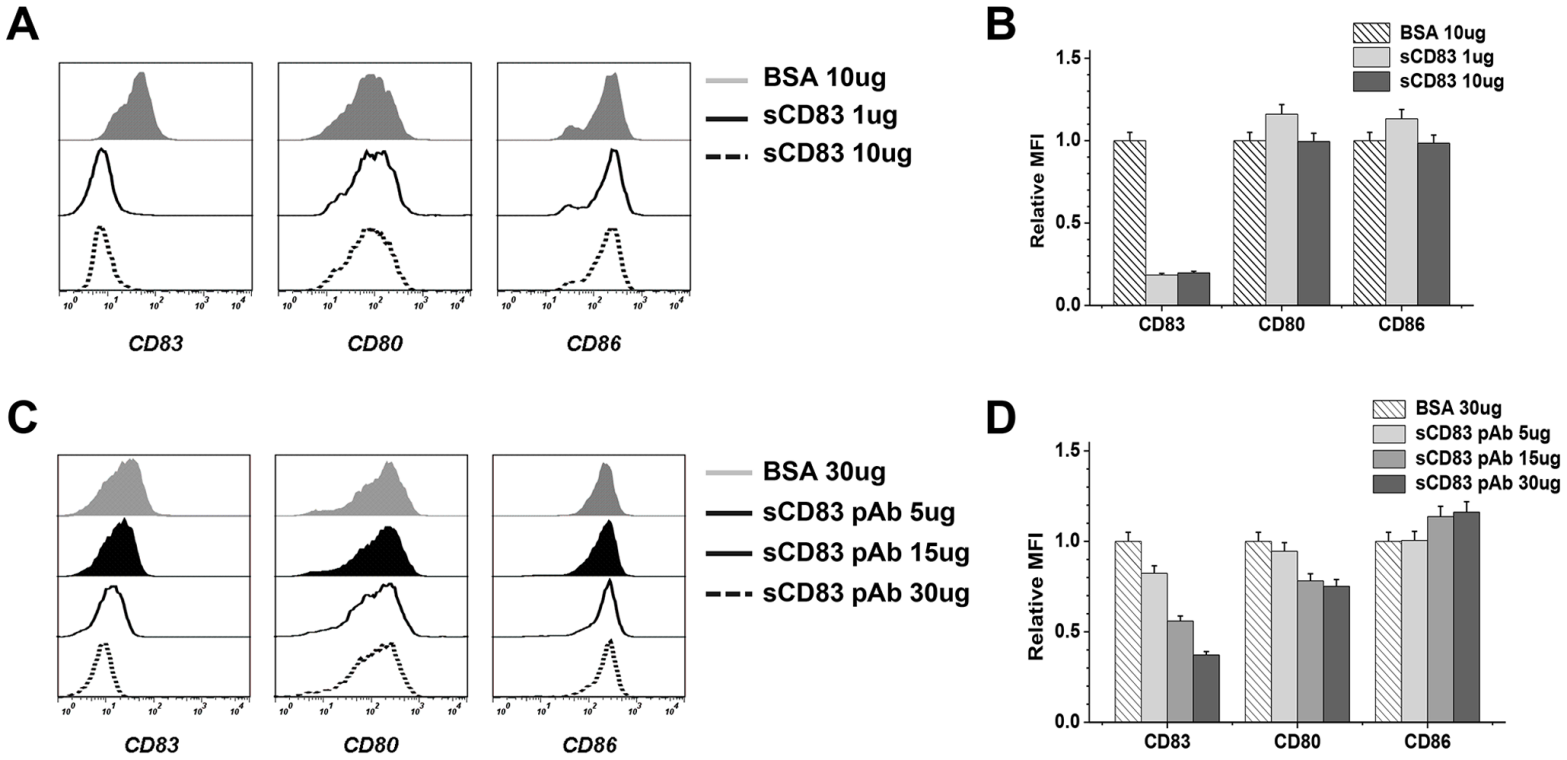
The specificity detection of sCD83. sCD83 has the similar epitope as the membrane form. (A, B) FACS competition tests of sCD83. Mature DCs were coated with the indicated antibody for DC surface molecules, and the indicated concentration of sCD83 was added. Representative FACS histograms are shown. (B) The data are from (A) an experiment that is representative of at least 3 independent experiments. (C, D) FACS competition tests of an anti-sCD83 pAb. Mature DCs were coated with the indicated antibody for DC surface molecules, and the indicated concentration of sCD83 polyclonal antibody was added. Representative FACS histograms are shown. (D) The data are from (C) an experiment that is representative of at least 3 independent experiments. The means ± the standard error of the mean (SEM) of the MFI are shown (B, D).

To detect the specificity of sCD83 and the anti-sCD83 polyclonal antibody (pAb) from immune rabbit serum, an antibody competition assay was performed. Mature DCs, which have high membrane CD83 expression levels, were stained with the commercial antibody after recombinant sCD83 was added to block the combination. We found that sCD83 from yeast could specifically block the binding of the commercial CD83 antibody to native CD83 on mature DCs but did not influence other surface molecules, such as CD80 and CD86 (Fig. 5A and B). In another competitive binding assay, the rabbit anti-sCD83 pAb exhibited similar specificity when blocking the binding of the commercial anti-CD83 antibody to native CD83 on mature DCs (Fig. 5C and D).

### The Binding of sCD83 to the Putative CD83 Receptor

**Figure 6:**
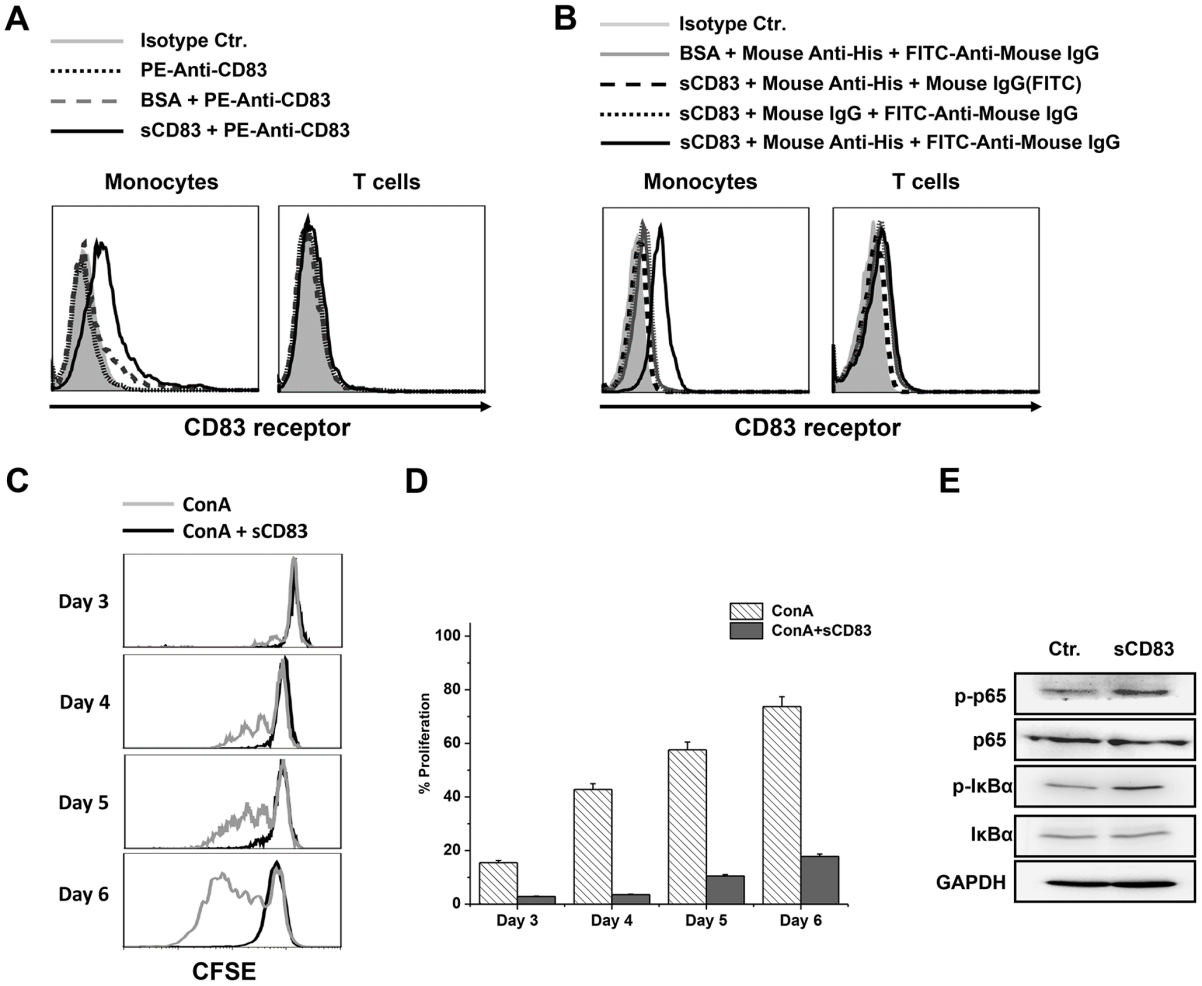
Biological activity determination of sCD83. sCD83 bound to the putative CD83 receptor on monocytes but not on T cells. (A and B) PBMC were incubated with soluble CD83 or BSA, and then coated with either PE-anti-CD83 (A) or anti-His followed by FITC-Anti-mouse IgG (B). Cells were then coated with anti-CD3 or anti-CD14 to distinguish the monocyte and T cells. CD14^+^ monocytes and CD3^+^ T cells were gated and analysis for CD83 receptor expression. (C) sCD83 suppressed ConA-stimulated PBMC proliferation. PBMCs were stained with CFSE, stimulated with ConA, and cultured with or without 20 µg/mL of sCD83 for the indicated time periods. (D) The means ± the standard error of the mean (SEM) of the MFI from (C) are shown as bar graphs. (E) sCD83 activated the NF-κB pathway. Isolated human PBMCs stimulated by sCD83 (10 µg/mL) were cultured in RPMI 1640 medium that contained 10% fetal bovine sera at 37°C for 12 h. The PBMCs were collected and lysed to detect the activation of the NF-κB pathway using Western blotting.

The putative CD83 counter receptor is expressed predominantly on monocytes [24]. To detect whether sCD83 from yeast could bind to the putative CD83 receptor, this receptor was screened on monocytes and T cells using flow cytometry. In freshly isolated PBMCs, sCD83 was found to mainly bind to CD14^+^ monocytes but not CD3^+^ T cells (Fig. 6A and B). sCD83 from yeast could specifically bind to its putative CD83 counterpart on monocytes, which indicated that sCD83 could be used as bait for the putative CD83 receptor.

### sCD83 Biological Activity Assay

Recombinant sCD83 exhibits significant immunosuppressive properties [15], [18]–[21]; therefore, we investigated the biological activity of sCD83 by suppressing ConA-stimulated PBMC proliferation *in vitro* as previously reported [28]. CFSE-labeled PBMCs were stimulated with ConA. Recombinant sCD83 was added to the culture systems for 3–6 days, and the proliferation of PBMCs was measured by flow cytometry. As shown in Fig. 6C and D, sCD83 can strongly inhibit ConA-stimulated human PBMC proliferation, which may be due to the binding of sCD83 to its putative receptor on monocytes. Then, we purified the monocytes from PBMCs and found that sCD83 from yeast could activate the NF-κB pathway through the phosphorylation of NF-κB p65 and IκBα (Fig. 6E), which indicated that the activation of the NF-κB signaling pathway and its downstream molecules may be responsible for the immunosuppression.

## Discussion

In this study, we first achieved the successful expression and purification of sCD83 from *P. pastoris* through fermentation at an extremely high cell density. The coding sequence for human sCD83 was subcloned into the pPIC9K plasmid, and the colonies that produced high levels of secreted sCD83 were screened and selected for further study.

To obtain the basic fermentation parameters for temperature and pH, the sCD83 expression conditions were optimized in flasks. In the temperature screening, the higher level of expression of sCD83 was obtained when it was cultured at 25°C, while significant reductions of total expression levels and the proportion of glycosylated sCD83 were observed at 20°C cultures, suggesting that the culture temperature has a great impact on the intensity of protein glycosylation modifications in yeast. We observed higher expression levels of sCD83 at pH 5.0 or 6.0 than at pH 4.0. After the optimal expression conditions were determined, we used a simple and common 2-phase fed-batch cultivation process to express sCD83, which was composed of a glycerol batch and a methanol feeding strategy that was maintained at a constant speed. This robust cultivation strategy resulted in the highest biomass density of sCD83 and the highest yield of sCD83 to date in the fermentation of *P. pastoris.*

sCD83 was simply purified using common His-Select affinity chromatography and size-exclusion chromatography to a purity of approximately 95%. After a 2-step purification process, we obtained purified sCD83 that had two isoforms with different molecular weights in 15% SDS-PAGE. sCD83 is an N-glycosylated protein with three potential N-glycosylation sites, which often lead to high mannose glycosylation in *Pichia pastoris*. Treatment with PNGase F decreased the molecular weight of sCD83 from yeast, which revealed that sCD83 was glycosylated. These results are in agreement with our hypotheses and previous reports. The possible glycan structure of sCD83 needs to be characterized in further studies. Furthermore, sCD83 was mainly expressed as a monomer, which was consistent with the results of the eukaryotic expression of sCD83 [27] but not consistent with the study by Lechmann *et al*. in which sCD83 was expressed in *E. coli* [30]. Eukaryotic microorganisms, such as yeast and mammalian cells, are more potent in post-translational modifications than *E. coli*. Therefore, it is could be speculated that native sCD83 expressed from DCs is mostly in the monomer form, whereas the dimer form of sCD83 that was expressed in *E. coli* may be the products from mismatched disulfides during the renaturation process.

An anti-sCD83 pAb was generated by immunizing rabbits with purified sCD83 that was produced from our yeast expression system. The rabbit anti-sCD83 pAb specifically bound to native CD83 on human monocytes derived DCs in a competitive manner with commercial anti-CD83 antibodies. Therefore, sCD83 that was generated by our method exhibited a similar structure as native sCD83 according to antigen-induced antibody production and binding specificity.

The ligand of CD83 remains unknown. To detect the CD83 ligand on monocytes, a modified sandwich fluorescence-activated cell sorter (FACS) assay with sCD83 and PE-CD83 antibodies was developed and employed in this study. The results revealed that sCD83 was able to specifically bind to its putative counterpart that was presented on the surface of monocytes, which confirms that the sCD83 that was produced in this study may possess the same confirmation structure as native CD83. In addition, the ligand of CD83 is a membrane protein that is presented on monocytes.

CD83 is a cell surface molecule with a unique pattern of expression and has multiple functions in suppressing immune responses and activating the NF-κB signaling pathway. In this study, the sCD83 that was expressed in *P. pastoris* was able to inhibit the proliferation of PBMCs from human volunteers and activate NF-κB, which was consistent with the observations in a recent report [28] and suggested that the sCD83 from yeast is an active form. These results suggest that fermentation in *P. pastoris* provides a sound strategy for the large-scale production of functionally active sCD83 for basic research and clinical applications.
